# Optimizing home-based long-term intensive care for neurological patients with neurorehabilitation outreach teams – protocol of a multicenter, parallel-group randomized controlled trial (OptiNIV-Study)

**DOI:** 10.1186/s12883-022-02814-y

**Published:** 2022-08-04

**Authors:** Thomas Platz, Thomas Kohlmann, Steffen Fleßa, Bernadette Einhäupl, Martha Koppelow, Lina Willacker, Hans-Jürgen Gdynia, Esther Henning, Jürgen Herzog, Friedemann Müller, Dennis A. Nowak, Romy Pletz, Felix Schlachetzki, Tobias Schmidt-Wilcke, Michael Schüttler, Andreas Straube, Rebekka Süss, Volker Ziegler, Andreas Bender

**Affiliations:** 1 Neurorehabilitation Research Group, Faculty of Medicine, University of Greifswald, Universitätsmedizin Greifswald, Fleischmannstrasse 44, 17475 Greifswald, Germany; 2 Institute for Neurorehabilitation and Evidence-Based Practice, “An-Institut”, BDH-Klinik Greifswald, University of Greifswald, Greifswald, Germany; 3 Institut für Community Medicine, Abt. Methoden der Community Medicine, Universitätsmedizin Greifswald, Greifswald, Germany; 4 Lehrstuhl für Allgemeine Betriebswirtschaftslehre und Gesundheitsmanagement, University Greifswald, Greifswald, Germany; 5 Department of Neurology, University Hospital, Ludwig-Maximilians-University Munich, Munich, Germany; 6 m&i-Fachklinik Enzensberg, Füssen, Germany; 7 Schön Klinik München Schwabing, Munich, Germany; 8 Schön Klinik Bad Aibling Harthausen, Bad Aibling, Germany; 9 VAMED Klinik Kipfenberg, Kipfenberg, Germany; 10 Klinik für Neurologie der Universität Regensburg am Medbo Bezirksklinikum, Zentrum für Vaskuläre Neurologie und Intensivmedizin, Regensburg, Germany; 11 Neurologisches Zentrum am Bezirksklinikum Mainkofen, Deggendorf, Germany; 12 Schön Klinik Bad Staffelstein, Bad Staffelstein, Germany; 13 Klinik für Neurologische Frührehabilitation, Rhön-Klinikum, Bad Neustadt a. d. Saale, Germany; 14 Therapiezentrum Burgau, Hospital for Neurorehabilitation, Burgau, Germany

**Keywords:** Neurorehabilitation, Weaning, Clinical trial, Protocol, Healthcare

## Abstract

**Background:**

Even with high standards of acute care and neurological early rehabilitation (NER) a substantial number of patients with neurological conditions still need mechanical ventilation and/or airway protection by tracheal cannulas when discharged and hence home-based specialised intensive care nursing (HSICN). It may be possible to improve the home care situation with structured specialized long-term neurorehabilitation support and following up patients with neurorehabilitation teams. Consequently, more people might recover over an extended period to a degree that they were no longer dependent on HSICN.

**Methods:**

This healthcare project and clinical trial implements a new specialised neurorehabilitation outreach service for people being discharged from NER with the need for HSICN. The multicentre, open, parallel-group RCT compares the effects of one year post-discharge specialized outpatient follow-up to usual care in people receiving HSICN. Participants will randomly be assigned to receive the new form of healthcare (intervention) or the standard healthcare (control) on a 2:1 basis. Primary outcome is the rate of weaning from mechanical ventilation and/or decannulation (primary outcome) after one year, secondary outcomes include both clinical and economic measures. 173 participants are required to corroborate a difference of 30 vs. 10% weaning success rate statistically with 80% power at a 5% significance level allowing for 15% attrition.

**Discussion:**

The OptiNIV-Study will implement a new specialised neurorehabilitation outreach service and will determine its weaning success rates, other clinical outcomes, and cost-effectiveness compared to usual care for people in need for mechanical ventilation and/or tracheal cannula and hence HSICN after discharge from NER.

**Trial registration:**

The trial OptiNIV has been registered in the German Clinical Trials Register (DRKS) since 18.01.2022 with the ID DRKS00027326.

## Background and objectives

### Background

A considerable proportion of patients with acute neurological conditions, such as stroke or traumatic brain injury (TBI), suffer from persistent disorders of consciousness (DoC), deficits of autonomic drive for breathing, severe paresis, and/or dysphagia (deficits of swallowing functions), severe enough to necessitate tracheostomy and use of a so called blocked tracheal cannula (preventing aspiration and aspiration pneumonia) and/or mechanical ventilation [1, 2]. Similarly, patients with conditions requiring prolonged intensive care unit treatment including ventilation support frequently acquire secondary neurological deficits of the central and peripheral nervous system as well as muscles with motor, sensory, and cognitive deficits and frequently emotional disorders, a syndrome called Post Intensive Care Unit Syndrome, PICS [3, 4].

Neurological early rehabilitation (NER) is the treatment of choice for this group of patients, promoting stabilization of organ function by intensive care treatment while at the same time reducing functional deficits based on an interdisciplinary rehabilitation team approach [5]. This type of treatment is rather successful. In a German cohort, 26% of 754 patients were mechanically ventilated at the time of admission to NER; their rate of weaning from mechanical ventilation was 65% during inpatient care in NER [6]. More recently, 36 weaning units in early neurological rehabilitation from 11 federal states in Germany with a total of 496 beds participated in another survey [7]. From 2516 weaning cases documented in 2019, 2097 (83.3%) could primarily be successfully weaned from mechanical ventilation and only 120 (4.8%) had to be discharged with home ventilation support.

Furthermore, a recent Germany-wide survey documented considerable hospital capacities (“beds”) for prolonged weaning from a mechanical ventilator for patients with neuro-disabilities [8]. Sixty-eight institutions declared to have such capacities with a broad distribution across Germany and its federal states. Overall, 1094 “beds” for prolonged weaning (and neurorehabilitation) were reported.

While effective treatment and capacities for such specialised healthcare is available in Germany (and other countries), there is still a substantial proportion of patients, who need mechanical ventilator support and/or airway protection by a (blocked) tracheostomy tube at discharge from neurological early rehabilitation. These patients will then need continuous (24/7) home-based specialized intensive care nursing with skilled staff being present to deal with any medical problems that might arise and need instantaneous reaction. In Germany, this form of out-of-hospital intensive care support can be provided in the patient´s home, in specialized long-term care facilities, or in shared apartments, where up to 6 patients can be cared for.

Given the demographic changes of societies and progress in survival rates of people receiving prolonged intensive care unit (ICU) treatment, the absolute numbers of people with need for home-based specialized intensive care nursing is rising considerably. Indeed, the number of people in need for invasive long-term ventilation in Germany dramatically increased over the past 15 years to an estimate of 20.000 patients in 2019, implying additional healthcare cost of around 4 billion Euros per year [9].

### Objectives and hypotheses

Neurological conditions frequently take a long time to recover and recovery can be promoted for prolonged periods by trained staff from various medical disciplines, e.g. medical doctors, therapists from various professions, and nurses trained in neurorehabilitation, most efficiently organized as interdisciplinary teams [10].

Inpatient NER is effective and beneficial for many neurological patients, because it has the key element of interdisciplinary, goal-oriented, and frequently adapted therapy and monitoring within a multi-professional team that is in constant communication [11, 12]. Long-term community-based care of neurological patients is often lacking these key elements of successful patient-centered care: involved and experienced physicians, nursing staff, and therapists are often highly motivated and provide excellent care, yet there is little coordination and communication between these different stakeholders and a lack of common patient-centered goals. A multi-professional team approach and combining efforts to improve functioning, activities, and participation seem, however, to be crucial for long-term outcome in neurorehabilitation [11, 12].

In such an situation, it might well be that if the sectorial healthcare borders and hence barriers for specialized long-term treatment were overcome that more people might recover over an extended period to a degree that they were no longer dependent from mechanical ventilation and were no longer in need for airway protection by blocked tracheal cannulas (via tracheostomy). If that could be achieved, the number of people requiring HSICN could be reduced.

This healthcare project and clinical trial “OptiNIV” (acronym for optimizing post-discharge intensive care for neurological patients) is set forth to:

1. Implement specialized post-discharge follow-ups by out-patient neurorehabilitation teams based at specialized neurorehabilitation hospitals for people with a long-term need for mechanical ventilation and/or tracheal cannula and hence for HSICN in the federal state of Bavaria (Germany)
2. Provide individualized managed care and treatments plans tailored to improve neurological functioning to healthcare professionals in charge at the community level
3. Implement structured short inpatient assessments at neurorehabilitation hospitals to identify patients, who are ready to be weaned
4. Implement a period of secondary inpatient rehabilitation to achieve weaning from mechanical ventilation and/or tracheostomy tube in patients assessed to have this potential
5. And to test with a parallel-group randomised controlled trial, RCT the superiority hypothesis that such a specialized outreach healthcare service increases the rates for weaning from mechanical ventilation and/or decannulation (and promotes other clinical and economic outcomes) within the first year post discharge from neurological early rehabilitation.

## Methods

### Trial design and characteristics

#### Design

This superiority trial is a multicentre, open, parallel-group RCT, which compares post-discharge specialized outpatient follow-ups by neurorehabilitation teams to usual care for people with a need for mechanical ventilation and/or tracheal cannula and hence for HSICN after discharge from NER. The RCT has the objective to assess effects of the specialized outreach healthcare service on rates for weaning from mechanical ventilation and/or decannulation and other clinical and economic outcomes within the first year post discharge.

#### Settings and locations

**Fig. 1:**
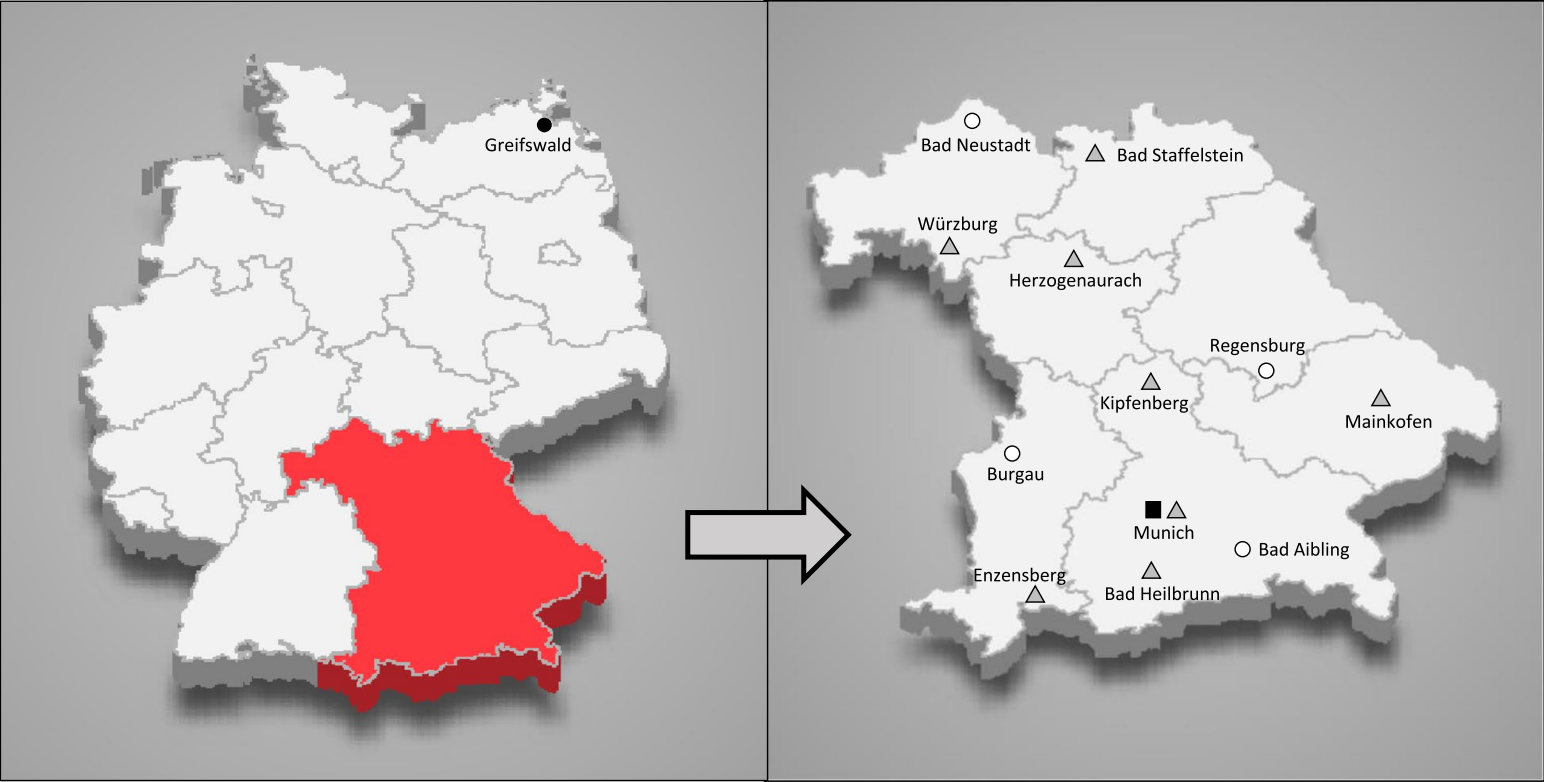
The 12 participating centres of the trial, the study coordination centre in Munich, and the scientific evaluation centres in Greifswald. ■ Study coordination centre: Department of Neurology, University Hospital, Ludwig-Maximilians-University Munich. ○ Regional Outreach Centres, ROC: Schoen Clinic Bad Aibling Harthausen; Therapy Centre Burgau; Clinic for Neurology of the University of Regensburg at medbo district hospital; Clinic for Neurological Early Rehabilitation/Intensive Care, Rhön-Klinikum, Campus Bad Neustadt. △ Participating Neurological Early Rehabilitation centre, NER: Juliusspital Würzburg, VAMED Klinik Kipfenberg, Schoen Clinic Bad Staffelstein, m&i Fachklinik Enzensberg, m&i Fachklinik Herzogenaurach, m&i Fachklinik Bad Heilbrunn, Neurological Centre at the Bezirksklinikum Mainkofen. ● Independent scientific evaluation centres in Greifswald: Greifswald University Medical Center; University of Greifswald. Source maps offered by Maxim Grebeshkov were obtained from Inmagine Lab Pte. Ltd ("123RF") with a license to use and adapt it

The study is implemented by a network of 12 cooperating centres of neurological early rehabilitation in the federal state of Bavaria (Germany) (compare Fig. 1) recruiting eligible patients at discharge from their service. These 12 centers provide full coverage of the population of approximately 13 million in Bavaria (www.statistikdaten.bayern.de).

Four of these centres provide the regional post-discharge specialized outpatient follow-ups with neurorehabilitation teams and constitute the “regional outreach centres” (ROC), resulting in a division of the federal state into four regions (North, East, South, West). Each ROC is responsible to establish the new healthcare service in its region.

Another study setting is the participants’ residences, where they receive the HSICN.

#### Trial status

Participant recruitment was planned to start in 2022. At the time of submission of this manuscript, the first patients have been enrolled.

### Participants

#### Eligibility criteria

Subjects aged 18 years or older with acute neurologic conditions (onset < 6 months ago), who are transferred to HSICN after completion of inpatient NER and who are still mechanically ventilated and/or cannulated will be included. When post-discharge weaning from mechanical ventilation and/or decannulation appears to be medically precluded or when life expectancy is less than 12 months candidates are not eligible for the trial. The detailed inclusion and exclusion criteria are given in Table 1.

**Table 1:** Inclusion and exclusion criteria

Inclusion criteria
1. Age ≥ 18 years
2. Health insurance with AOK Bayern
3. Inpatient neurological early rehabilitation with a neurological rehabilitation diagnosis
4. Mechanical ventilation and/or tracheal cannula
5. (Sub-)Acute medical condition (onset < 6 months before study entry)
6. Planned discharge to home-based intensive care
7. Informed consent for study participation
**Exclusion criteria**
1. Palliative treatment/life expectancy < 12 months (according to medical assessment)
2. Weaning/decannulation prospectively medically excluded (e.g., high cervical paraplegia, laryngeal tumor)
3. Preexisting home-based intensive care
4. Progressive neuromuscular disease (e.g., muscular dystrophy, ALS)
5. Home-based intensive care by a team already providing care to OptiNIV study participants

#### Screening

Screening will primarily take place in the 12 NER hospitals. The first patient was enrolled in March of 2022 and recruiting will continue through the middle of 2023. In addition to the hospital-based recruitment in participating centres, the AOK Bavaria (public health insurance) will support recruitment of eligible persons treated in and discharged from other hospitals in Bavaria.

#### Recruitment and baseline assessment

After assessing eligibility, detailed information will be provided to the patient and/or his/her legal guardian by physicians of the 12 NER hospitals, and written consent to study participation will be obtained ("informed consent"). Afterwards, the baseline visit (t_0_) will take place at the NER hospital prior to discharge to HSICN. As part of this, demographic and clinical data are collected as well as information on the participant’s mechanical ventilation and tracheostomy status, use of other medical devices, health status and quality of life by using questionnaires and checklists (Table 2). The visit will be carried out by trained site personnel and the results will be recorded in case report forms (CRF).

**Table 2:** Outcome measures and assessment schedule

		**Study period**					
		**t**_**0**_	**t**_**1**_	**t**_**2**_	**t**_**3**_	**t**_**4**_	**t**_**5**_
**Outcome**	**Assessment tool**	**Baseline**	**Month 1**	**Month 3**	**Month 6**	**Month 9**	**Month 12**
Status tracheal cannula (primary)	Decannulated [Y/N] [Reasons/Checklist) Unblocking time/day [min.] Total duration	x	x	x	x	x	x
Status mechanical ventilation (primary)	Weaned [Y/N] Current weaning stage Total duration	x	x	x	x	x	x
Survival	Death [Y/N] Time to death after discharge		x	x	x	x	x
Medical devices (e.g. PEG tube)	Checklist	x	x	x	x	x	x
Specific neurological medication	Checklist	x	x	x	x	x	x
Global neurological outcome	Glasgow Outcome Scale Extended (GOS-E)	x	x	x	x	x	x
Neurological status: Level of consciousness Degree of paresis Spasticity Dysphagia	Coma Recovery Scale-Revised (CRS-R) Motricity Index (MI) Resistance to Passive Movement Scale (REPAS) Bogenhausener Dysphagie Score (BODS) Penetrations-Aspirations Skala (PAS)	x	x	x	x	x	x
Pain scale	Visual analogue scale (0 – 100)	x	x	x	x	x	x
Activities of daily life	Barthel-Index (BI)	x	x	x	x	x	x
Quality of life (QOL) (Patient and relatives)	European Quality of Life-5 Dimensions (EQ-5D-5L)	x	x	x	x	x	x
Social participation (Patient and relatives)	WHO Disability Assessment Schedule (WHODAS 2.0)	x					x
Depression/anxiety (Patient and relatives)	Hospital Anxiety and Depression Scale (HADS)	x	x	x	x	x	x
Burdens on the relatives (relatives)	Modified Caregiver Strain Index (MCSI)		x	x	x	x	x
Complications (including unplanned hospitalisations)	Semi-standardised free text	x	x	x	x	x	x
Satisfaction with health care situation (Patient, relatives, and home-based intensive care personnel)	10-point Likert scale		x	x	x	x	x
Adherence to the treatment pathway (IG)	[Y/N] Checklist Free text		x	x	x	x	x
Utilisation of healthcare (e.g. HSINC, hospital stays, outpatient medical services, aids)	Checklist AOK Bavaria data		x	x	x	x	x
Costs at the expense of SHI (separated according to sectors, cost types)	Costs per type in Euro [€] (AOK Bavaria)						x
Whereabouts of the patient	Checklist		x	x	x	x	x

*IG* Intervention group, *SHI* Statutory health insurance

### Intervention

#### Experimental condition

The experimental condition is a new systematic healthcare bundle, primarily based on post-discharge specialized outpatient follow-ups by ROC-based neurorehabilitation teams for people with a need for ongoing mechanical ventilation and/or tracheal cannula and hence for HSICN after discharge from NER. The intervention will provide team-based neurorehabilitation healthcare counselling and support during the first year after discharge from NER that aim at promoting further functional neurological recovery with a focus on breathing and swallowing and consecutively continued weaning from mechanical ventilation and/or promoting a functional status that safely allows decannulation. Teams are physician-led and can include specialized breathing therapists trained to support weaning form mechanical ventilation, dysphagia therapists, physiotherapists, occupational therapists, and/or specialized nursing staff.

Elements of the healthcare bundle are:

1. Team sessions in the ROC to evaluate participants’ data (see 2 & 3), develop and communicate a managed care plan with healthcare recommendations to the HSICN staff, treating physicians and therapists;
2. Continuous exchange of vital data, mechanical ventilation and tracheal cannula management data, as well as laboratory data between the HSICN and the ROC; a telephone hotline is available to HSICN in case of need for advice regarding patient care;
3. Regular patient visits within their HSICN-setting by the ROC-based outreach teams to assess participants’ clinical status personally including blood gas analysis, BGA, and functional swallowing assessment (fiberendoscopic evaluation of swallowing, FEES);
4. Short inpatient re-evaluations in the ROC when a potential for weaning and/or decannulation is observed;
5. Inpatient neurorehabilitation period (couple of weeks) in the ROC when the short inpatient re-evaluation indicated the likelihood of a successful weaning and/or decannulation.

Sequence of events:

As a central part of the experimental intervention, therapy plans for long-term weaning from mechanical ventilation and/or decannulation will be developed in collaboration with outpatient healthcare providers (e.g. general practitioners, physiotherapists, HSICN staff).

Defined changes of vital signs will trigger the HSICN staff to send the current patient's medical condition reports to the ROC. Based on these reports, the therapy plans and weaning protocols will be updated in interdisciplinary team conferences and will be forwarded to the home-based intensive care staff.

In addition, the intervention includes visits to study participants by outpatient follow-up teams consisting of physicians and therapists dispatched by the ROC. Each ROC has an outpatient follow-up team that covers its region. The visits will start 4 weeks after discharge from neurological early rehabilitation and will continue with visits every three months thereafter for one year. At each visit (t_1_-t_5_) the outpatient follow-up teams perform discipline-specific examinations, including blood gas analysis (BGA) and FEES. Additional visits to the HSICN by the ROC are possible, if needed.

Once the potential for weaning from mechanical ventilation and/or decannulation is considered by the outpatient follow-up team, the study participant will be admitted to the respective ROC. Here, a more comprehensive assessment of the potential for weaning from mechanical ventilation and/or decannulation is conducted as part of a structured diagnostic process over a few days (“Interdisciplinary Inpatient Structured Assessment”, IISA).

If the potential for successful weaning and/or decannulation is confirmed during an IISA, an inpatient neurorehabilitation program with a weaning procedure similar to NER will follow (“Neurological interval rehabilitation”, NIR).

In addition to the above-mentioned experimental intervention aspects the outpatient follow-up teams will document study data at each visit (t_1_-t_5_) (as specified in Table 2).

For compensation to those who suffer harm from the experimental intervention a liability insurance coverage has been established.

#### Control condition

Participants in the control group will not receive trial-related healthcare interventions, but rather the conventional treatment, i.e. the standard healthcare as currently applied in the outpatient setting in Germany. In this setting, patients in the HSICN-setting are usually cared for by a general practitioner (and/or specialized physicians) and therapists as individually needed and prescribed. Aside from the specific experimental intervention (as described above) no concomitant care and interventions are prohibited in the control condition during the trial. The comparator was chosen since the research question is whether the introduction of the new healthcare practice affects outcome including cost-effectiveness; thus a comparison to “usual care” is warranted.

Participants in the control group do, however, also receive visits from the outpatient follow-up team at the same frequency as participants in the intervention group, but only for study data collection (compare Table 2).

**Fig. 2:**
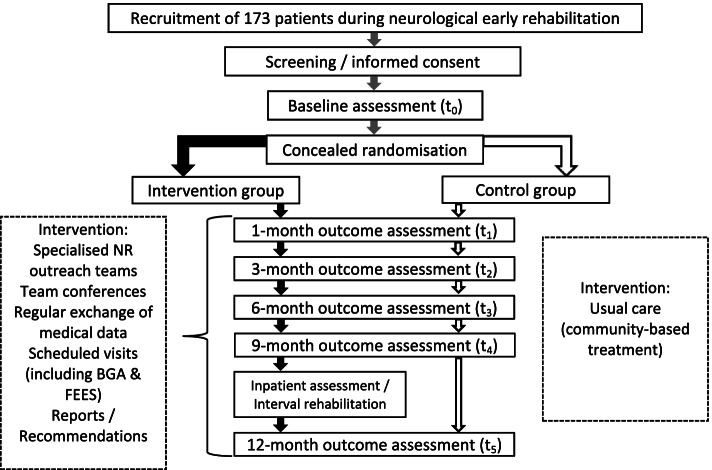
Flow chart: Trial course of control and intervention group in the OptiNIV trial. Abbreviations: NR = Neurorehabilitation; FEES = fiberoptic endoscopic evaluation of swallowing; BGA = blood gas analysis

All trial participants (experimental and control condition) will receive recommendations for post-trial care with their final visit Fig. 2.

### Outcomes

Outcomes of the trial are specified in Table 2.

#### Primary outcomes

The primary outcome is defined as the rate of participants, who could be weaned from mechanical ventilation and/or decannulated one year after discharge in the intervention and control group, respectively.

#### Secondary outcomes

Secondary outcomes include the following: Tracheal cannula and ventilatory status and modalities, mortality, neurological status, activities of daily living, quality of life (QOL), depression/anxiety, pain, social participation, burden on the relatives, healthcare situation (use of home-based intensive care, living arrangement), satisfaction with healthcare situation, utilisation of healthcare (medical services, medication, remedies and aids, hospital care), complications/adverse events (including unplanned hospitalisation).

#### Outcome assessment

Outcome assessment will be performed by trained study personnel at visit t_0_—t_5_, with the first visit taking place at the original NER hospital before discharge, and the remaining visits conducted at participants’ home. Visiting participants in their homes will be feasible and promote participant retention and complete follow-up. Standardized and validated assessment instruments (e.g. questionnaires, checklists, scales) will primarily be used for this purpose. Some endpoints will also be collected from patients' relatives (QOL, social participation, depression/anxiety, caregiver burden, satisfaction with healthcare situation) and from home-based intensive care personnel (satisfaction with healthcare situation). Aside from medical management as indicated on a case by case basis, serious adverse events need to be reported by the documenting centre to the co-ordinating study centre for further evaluation and further action (e.g. meeting of the safety board, information of the institutional review board). Table 2 shows which data are gathered, at which point in time, using which assessment instrument, and from whom. Outcome data will be continued to be collected for participants who discontinue or deviate from the intervention protocol (experimental condition) as long as they consent to data collection.

#### Health economic analysis

In order to examine the cost-effectiveness of the intervention compared to standard healthcare, a health economic evaluation will be conducted as part of the project. Healthcare utilization and cost will be calculated individually using routine data provided by the public health insurance AOK Bavaria, data from questionnaires administered at t_5_, and interviews. Outcomes for the health economic analyses include (a.) utilisation and cost for hospital stays, medical doctors, therapists, medication, nursing, aids; (b.) cost for the experimental intervention; and (c.) health-related cost incurred by patients and relatives themselves.

#### Monitoring and data management

Before recruitment begins, the study centres receive training on the study protocol, use of the assessment instruments, the study documentation, the forwarding of the study documentation to the evaluating institutions, as well as the process and obligations regarding data validation. Video-based and written training material is made available via an access-restricted web site for continuous training purposes. Individual training and delegation of study processes are documented by corresponding logs in investigator site files.

The monitoring is carried out by the central project co-ordination (LMU) and the evaluating institution (UMG) independently of both the funding agency and participating centres and will commence after the inclusion of the first patients, in order to ascertain compliance with the study protocol, and to identify problems and difficulties in the implementation and documentation at an early stage.

After enrolment, patients will receive an identification number as an encryption (pseudonymisation), so that their names will only appear on the informed consent form and a patient identification log, but not on other study documentation. The data collection is realized on the basis of CRFs (Case Report Forms).

The CRFs are forwarded digitally from the recruiting centres or ROC to password-protected cloud areas on servers of the University Medical Center Greifswald responsible for the independent clinical scientific evaluation. The original CRFs remain in the recruiting centres or ROC, respectively.

Subsequently, the evaluating institution will review all CRFs for missing entries, inconsistencies and implausibility, and resolve potential queries with the study teams of the participating centres. Thereafter, CRF-documented trial data will be entered in a digital database and double-checked for correctness.

The AOK Bavaria transmits the pseudonymised data of inpatient treatment to the University of Greifswald (UG) for the health economic evaluation. A trustee agency is responsible for providing a linking between the primary data of the study (as documented on CRFs) and the routine data of the AOK Bavaria.

### Sample size

Based on prior studies [13], an event (success) rate of 30% in the intervention group and 10% in the control group after one year is estimated. Considering a 2:1 randomisation (intervention: control), a power of 80% and a significance level alpha of 5%, a study sample of 147 participants, with 98 patients in the intervention group and 49 patients in the control group is needed to corroborate an effect of the assumed magnitude statistically. Accounting for an expected dropout rate of 15% after one year the number of patients to be recruited is planned with 173 participants, 115 patients in the intervention group and 58 patients in the control group.

The strategies for achieving adequate participant enrolment to reach the target sample size had been based on number of cases being treated in the past in the participating centres who would have fulfilled the eligibility criteria, and planning of the recruitment period appropriately to allow for sufficient recruitment. Further, additional recruitment is planned via the participating healthcare insurance’s (AOK Bayern) case management that will identify potential participants from the region were the trial is conducted (federal state Bavaria, Germany) that were not seen by the participating recruiting centres.

### Randomisation

#### Sequence generation

Patients will be randomised on a 2:1 basis to receive the new form of healthcare (intervention) or the standard healthcare (control). Randomisation will be stratified for the 4 ROCs and the subgroups (a) with mechanical ventilation and (b) with tracheal cannula without mechanical ventilation. Patients will be block-randomised in blocks with varying length. Randomisation follows the baseline visit (t0), while patients are still in the neurological early rehabilitation centres.

#### Allocation concealment

Randomisation will be carried out externally by the UMG based on an online tool and randomization software in order to ensure a concealed, unpredictable allocation.

#### Implementation

The result of the andomization will be communicated to the recruiting centre, the ROC in charge, the evaluation centre, and the study coordination centre at the LMU (for recruitment monitoring purposes).

### Statistical analyses

#### Statistical methods used to compare groups for primary and secondary outcomes

The primary analysis will be performed as an intention-to-treat, ITT analysis.

Descriptive statistics will include relative frequencies, median and interquartile range (IQR), mean and standard deviation (SD), depending on the scale level. Relative risk or mean differences and 95% confidence intervals will be used to estimate intervention effects. Differences between the groups will be examined using multivariate regression methods. To analyse primary and secondary outcomes over time, generalised linear models (GLM) will be used for longitudinal data.

#### Methods for additional analyses

The sample size is calculated to test the primary hypothesis for the total study population with sufficient statistical power. It does not provide statistical power for subgroup analyses (assuming effects of a comparable magnitude). Therefore, the planned investigation of interaction effects (subgroup analyses) will only be exploratory.

Additional between-group analyses for secondary outcomes will be performed in an analogous way as stated for primary outcomes.

Associations between patient characteristics and outcomes, or among different outcomes will be analysed by correlational analyses.

Based on the outcomes documented for the health economic analysis utilization and cost comparisons between experimental and control group as well as outcome-related cost-effectiveness analyses will be performed analyzing the relationship between benefit (harm) and cost incurred.

In addition, system dynamics models will be used to assess potential future cost-effectiveness of the intervention for the post-intervention phase.

An interim analysis is not planned.

### Risk of bias considerations

The study design was chosen to ensure the best possible protection against systematic bias.

Potential selection bias is prevented by the concealed, stratified random allocation of patients to treatment groups in blocks by a partner not involved in recruitment.

No blinding of study personnel, patients or those conducting the evaluation will be possible for the experimental intervention, which increases the potential for performance bias and detection bias. Given the objective nature of the primary outcomes risk of detection bias is, however, considered low for these outcomes.

To reduce potential over- or underestimation of the intervention effect due to study dropouts (attrition bias), an intention-to-treat analysis will be conducted.

## Discussion

Even with highly effective neurological early rehabilitation, a substantial proportion of patients do still need mechanical ventilation and/or airway protection by a (blocked) tracheostomy tube at discharge [6, 7]. These patients need continuous (24/7) home-based specialized intensive care nursing after discharge from early rehabilitation with skilled staff being present to deal with any medical problems that might arise and need instantaneous reaction. While long-term recovery is possible with many neurological conditions (including stroke and PICS), healthcare systems (as in Germany) frequently don’t provide specialised interdisciplinary neurorehabilitation team treatment at the community level.

This healthcare project implements a structured clinical management with specialized post-discharge follow-ups by neurorehabilitation teams for this group of long-term neurological patients, providing individual managed care plans to healthcare professionals in charge at the community level, as well as short inpatient assessments or repeat inpatient rehabilitation stays when a potential for weaning from mechanical ventilation and/or decannulation is observed during follow-up.

The trial as described in this protocol will test the hypothesis that such a specialized outreach healthcare service increases the rate for weaning from mechanical ventilation and/or decannulation (and promotes other clinical and economic outcomes) within the first year post discharge from neurological early rehabilitation when compared to usual care.

The trial is set-up to provide the means to answer the research question of interest, to describe comprehensively effects of the new trans-sectorial reach-out healthcare service on medical status, emotional well-being, activities of daily living, and participation of patients being treated as well as caregiver well-being and burden.

In addition, the health economic analysis will provide estimates of cost-effectiveness comparing benefit (and harm) as well as cost (savings and expenditure) related to the new healthcare service both during the study period and a model how the intervention might affect future cost-effectiveness.

To summarize, the healthcare project and clinical trial will implement a new specialised neurorehabilitation outreach service for people being discharged from neurological early rehabilitation with the need for home-based intensive care nursing. The trial described in the protocol will comprehensively answer the research question whether and which clinically relevant benefits this service will generate. The integrated health economic evaluation will determine its cost-effectiveness.

## Data Availability

The study protocol is made available as open access publication in agreement with SPIRIT [14] and CONSORT [15] criteria. The final trial dataset will primarily be available for the independent evaluation centres (UMG and UG) and the study co-ordinating centre (LMU) as well as for participating centres on request. Data protection regulations restrict use of the trial data for purposes and by institutions being agreed on by participants providing written informed consent. Any queries regarding data availability can be forwarded to the corresponding author. The trial results will be made available by scientific publication and report to the funding agency. Authorship eligibility guidelines of publishing peer-review journals will be applied.

## Abbreviations

BGA: Blood gas analysis
CRF: Case report forms
DoC: Disorders of consciousness
FEES: Fiberendoscopic evaluation of swallowing
GLM: Generalised linear models
HSICN: Home-based specialised intensive care nursing
ICU: Intensive care unit
IQR: Interquartile range
ITT: Intention-to-treat
IISA: Interdisciplinary Inpatient Structured Assessment
LMU: Ludwig-Maximilians-University (Munich)
NER: Neurological early rehabilitation
PICS: Post intensive care syndrome
QOL: Quality of life
RCT: Randomized controlled trial
ROC: Regional outreach centre
TBI: Traumatic brain injury
UG: University of Greifswald
UMG: University Medical Center Greifswald
